# Remarks on the Apothecaries' Act

**Published:** 1815-12

**Authors:** 


### *For the London Medical and Physical Journal.*

### *Remarks on the Apothecaries' Act;* by An Apprentice.

In perusing your Journal for September, I was a little astonished to find apprentices articled previous to the new Act for the better Regulating the Practice of Apothecaries, so much affected by it; it will be the cause of great trouble and inconvenience to themselves and friends.

I am an apprentice to a surgeon-apothecary; the term specified in my indentures is four years; I was with an apothecary some time before any agreement could be drawn up, on account of my father's absence, he being nearly two hundred miles from this place. The term mentioned will expire early in the year 1818, when I shall have attained my 21st year, and intend devoting the remaining seven months, either with my worthy master, or attending some public institution, until the lectures commence. I went to school in Oxford, and the Grammar School in Giggleswick, until I was nearly seventeen years old, under the idea that four years would be sufficient, and the time expected of young men attending to pharmacy and surgery before they visit the university.

My master has two young men besides myself, articled for four years, one of whom intends to attend the second course of lectures, which I suppose commences early in 1816, but is involved in difficulties owing to the obnoxious part of the act alluded to, knowing that, without the said act is revised and corrected, he will not be allowed to undergo an examination, unless he puts his friends to unnecessary expence; having served a druggist fourteen months, and his late master four years and three months, I think it sufficient. I perfectly agree with my brother apprentice, who hinted his objection to this part of the act, which, if not taken into serious consideration by the members of the society, will make it advisable for us to petition for the amendment of the prime part of the act. We are perfectly aware, that it will be with extreme difficulty every young man can meet with a situation as assistant, &c. I shall be much gratified, should I see, in any of your future Numbers, that any one of the members has laid before the society an amendment, by which we may be relieved.

Before I conclude, allow me to express my own and fellow pupil's satisfaction with every other part of the act, deeming it a necessary mode for suppressing those charlatans which much much infest this country, and puff off their enormous cures, and impose upon a credulous public.

*October* 25, 1815.

The above letter so entirely coincides with our wishes, that we have not scrupled to insert it in the author's words. If men bred to the profession are to be scholars, the longer they remain at school the better; and, if they have certificates to shew that they have been four or five years after the age of sixteen or seventeen in an apothecary's shop, we conceive them fairly entitled to an examination; at least, we think the committee of examiners might be allowed a discretionary power on some such occasions.—EDIT.

